# Dexmedetomidine‐up‐regulated microRNA‐381 exerts anti‐inflammatory effects in rats with cerebral ischaemic injury *via* the transcriptional factor IRF4

**DOI:** 10.1111/jcmm.16153

**Published:** 2020-12-13

**Authors:** Hua Fang, Hua‐Feng Li, Jian‐Yong Yan, Miao Yang, Jian‐Ping Zhang

**Affiliations:** ^1^ Department of Anesthesiology Guizhou Provincial People’s Hospital Guiyang China; ^2^ Department of Anesthesiology Guizhou University People’s Hospital Guiyang China; ^3^ Laboratory of Anesthesiology & Perioperative Medicine Guizhou University School of Medicine Guiyang China; ^4^ Department of Anesthesiology West China Second University Hospital Sichuan University Chengdu China

**Keywords:** cerebral ischaemic injury, dexmedetomidine, IL‐9, IRF4, microRNA‐381, neuron cell apoptosis

## Abstract

Dexmedetomidine (Dex) possesses analgesic and anaesthetic values and reported being used in cerebral ischaemic injury therapeutics. Accumulating studies have determined the effect of microRNAs (miRNAs) on the cerebral ischaemic injury. Thus, the present study aimed to unravel the molecular mechanism of miR‐381 and Dex in cerebral ischaemic injury. For this purpose, the cerebral ischaemic injury rat model was established by induction of middle cerebral artery occlusion (MCAO) and expression of miR‐381 and IRF4 was determined. Thereafter, MCAO rats were treated with Dex, miR‐381 mimic, miR‐381 inhibitor and oe‐IRF4 respectively, followed by evaluation of neurological function. Furthermore, neuron cells were isolated from the hippocampus of rats and subjected to oxygen‐glucose deprivation (OGD). Then, OGD‐treated neuron cells and primary neuron cells were examined by gain‐ and loss‐of‐function assay. Neuron cell apoptosis was detected using TUNEL staining and flow cytometry. The correlation between interferon regulatory factor 4 (IRF4) and interleukin (IL)‐9 was detected. Our results showed down‐regulated miR‐38 and up‐regulated IRF4 in MCAO rats. Besides, IRF4 was targeted by miR‐381 in neuron cells. Dex and overexpressed miR‐381, or silenced IRF4 improved the neurological function and inhibited neuron cell apoptosis in MCAO rats. Additionally, in MCAO rats, Dex was found to increase the miR‐381 expression and reduced IRF4 expression to decrease the IL‐9 expression, which suppressed the inflammatory response and cell apoptosis both in vivo and in vitro. Importantly, our study demonstrated that Dex elevated the expression of miR‐381, which ultimately results in the inhibition of inflammation response in rats with cerebral ischaemic injury.

## 1 INTRODUCTION

The cerebral ischaemic injury, often induced by ischaemic stroke, is associated with long‐term disability.^1^ However, in more serious conditions, of cerebral ischaemic injury, inflammation, ischaemia and glial cell dysfunction contribute to persistent brain injury,^2^ thus indicating that dysfunction of glial cells and damage of the blood‐brain barrier are involved in ischaemic brain injury.^3^ Nonetheless, cerebral ischaemic injury has been attributed to the devastating complication of neurological and cardiovascular surgeries,^4^ while the neurodegenerative disorders caused by brain injury significantly impair the memory and learning ability, limb use, and other neurological performances.^5^

Moreover, dexmedetomidine (Dex) is an α2‐adrenergic receptor agonist which possesses analgesic and sedative properties.^6^ Intriguingly, Dex has been demonstrated to enhance the outcomes of the cardiac and neurological surgeries and to relieve the patient pain.^7^ Importantly, Dex has been reported to exhibit neuroprotective against cerebral ischaemic or reperfusion injury in rats.^8^ Besides, other studies have illustrated the protective effect of Dex on the ischaemic or reperfusion injury of various organs including the heart and kidney.^8^ On the other hand, recent studies have indicated the prognostic value of circulating miR‐19a and suggested it as a promising molecular target for early diagnosis and prognosis of acute myocardial infarction.^9^ Though microRNAs (miRNAs), that is miR‐129‐5p^10^ and miR‐29b,^11^ have indicated being involved in the neuroprotective effect of Dex. Yet, scarce studies investigated the relation between Dex and miR‐381 in cerebral ischaemic injury. Thus, the present study attempted to unravel the mechanism through which miR‐381 enhances the neuroprotective effects of Dex in cerebral ischaemic injury.

Importantly, miR‐381 has been demonstrated to possess a crucial role in the proliferation and differentiation of neural stem cells.^12^ Further studies have proved that miR‐381 restores the injury of cerebral ischaemia in rats and exerts neuroprotective function.^13^ Moreover, the expression of mmu‐miR‐381 could be elevated after Dex treatment in lung injury.^14^ Noticeably, a close relationship has been reported between miRNA and interferon regulatory factor (IRF) in the brain. For instance, miR‐301 associated with IRF1 was indicated being implicated in the neuronal innate immune response.^15^ Additionally, further studies have illustrated IRF4 as a transcriptional factor correlated with the inflammatory response in the brain and suggested it as a target for the injury of neonatal ischaemic brain.^16^

Thus, in the present study, we hypothesized that miR‐381 and IRF4 might have an association with a Dex‐mediated protective effect against cerebral ischaemic injury in rats. Hence, our study aimed to investigate the impacts of Dex‐regulated miR‐381 and the relevant regulatory mechanism in cerebral ischaemic injury.

## 2 METHODS AND MATERIALS

### 2.1 Ethics statement

The study was conducted with the approval of the Animal Ethics Committee of Guizhou Provincial People's Hospital. All rats were treated by following the *Guidelines for the Care and Use of Laboratory Animals* proposed by the National Institutes of Health.

### 2.2 Bioinformatics analysis

The downstream target genes of hsa‐miR‐381 were predicted by online websites StarBase,^17^ mirDIP,^18^ miRWalk^19^ and TargetScan.^20^ Human transcription factor names obtained from the Cistrome database.^21^ The target genes of rno‐miR‐381 were obtained by miRDB (Target score ≥ 60),^22^ miRWalk (Top 500 with the accessibility of binding relation) and TargetScan (Cumulative weighted context ++ score ≤ −0.05). In order to obtain more reliable target genes, Venn mapping was carried out to obtain the common genes predicted by online tools.

### 2.3 Establishment of rat models

A total of 120 specific pathogen‐free Sprague‐Dawley rats (males; aged 3 months) were used in this study. Among them, 12 rats underwent sham‐operation and 108 rats were used for model establishment. The MCAO model was established by following the previously reported procedures at room temperature (20 ± 5°C).^13^, ^23^ The five‐point scale (Longa scoring system) introduced in previous work was utilized for the evaluation of the modelling,^24^ that is 0 point: no neurological deficit; one point: unable to fully extend left forepaw; two points: circling to the left; three points: difficult to walk and leaning to the opposite side; and four points: unable to walk spontaneously and almost lost consciousness. The rats with score 0 and 4 were excluded from this study, while rats with score 1‐3 were included.

### 2.4 2,3,5‐Triphenyltetrazolium chloride (TTC) staining

TTC staining was used to observe the infarcted area.^25^ The rats were anaesthetized 24 hours after operation. The brain tissues were cut into 2‐mm‐thick sections and stained with 2% TTC (G3005, Solarbio, Shanghai, China) at 37°C for 30 minutes. After fixation with 4% paraformaldehyde for 2 hours at 4°C, the slices were photographed. The ischaemic part of the brain tissues was white, while the normal brain tissues were red or purple. The area of cerebral infarction was calculated.

After MCAO modelling, five rats were randomly selected and the brain tissues were removed. The area of cerebral infarction was detected by TTC staining to evaluate the success of MCAO modelling. TTC staining and infarct size calculation showed that MCAO rat model establishment was successful (Figure S1A, B).

### 2.5 Rat grouping

As shown in Figure S2, the rats were grouped into nine groups (12 each). Before MCAO modelling, the lateral ventricle of the rats was injected with artificial cerebrospinal fluid containing miR‐381 inhibitor, inhibitor negative control (NC), miR‐381 mimic, mimic NC, miR‐381 mimic + lentivirus‐packaged overexpression (oe)‐NC or miR‐381 mimic + lentivirus‐packaged oe‐IRF4 at 0.6 mg/kg, or injected with Dex or 0.9% normal saline at 50 µg/kg (30 minutes prior to MCAO), respectively.

### 2.6 Primary hippocampal neuron cell culture and oxygen‐glucose deprivation (OGD) treatment

The primary neuron cells were isolated from the post‐natal mice (n = 3) by following the instructions of Papain dissociation kit (#3150; Worthington Chemicals), followed by isolation of mixed neuron cells from the rat hippocampal tissues according to the protocols of the manufacturer. Then, cells were cultured and grown to confluence according to the detailed procedure established before.^26^ Subsequently, the primary neuron cells for OGD treatment were cultured in serum‐ and glucose‐free medium and then transferred into a box with 95% N_2_ and 5% CO_2_ for 6‐hours incubation, while the normal control was placed in norm‐oxygenated DMEM containing glucose.

### 2.7 Cell grouping

Lentivirus vector LV5‐GFP (#25999; Addgene Inc) was adopted for overexpression, while pSIH1‐H1‐copGFP (LV601B‐1; System Biosciences) was utilized for silencing of cells. The cells were transfected with short hairpin (sh)‐RNA targeting IRF4, sh‐NC, miR‐381 inhibitor, and the corresponding inhibitor NC separately or combinedly constructed by Shanghai GenePharma Co., Ltd.. Afterwards, the packaged virus and targeted vectors were cotransfected into HEK293T cells and incubated for 48 hours followed by the collection of supernatants. Then, viral particle was filtered by the centrifugation of supernatant and virus titre was determined. Following after, the HEK293T cell was cultured in RPMI‐1640 medium containing 10% FBS and subcultured after every 2 days, and the virus in the exponential growth period was obtained. Meanwhile, control cells were treated or untreated with sh‐NC lentivirus, sh‐IRF4 lentivirus, oe‐NC lentivirus, oe‐IRF4 lentivirus or Dex, while OGD‐treated cells were treated or untreated with Dex, oe‐NC lentivirus + Dex, oe‐IRF4 lentivirus + Dex, inhibitor NC lentivirus + Dex or miR‐381 inhibitor lentivirus + Dex. Subsequently, cells were cultured at 37°C in 5% CO_2_ for 48 hours.

### 2.8 Modified neurological severity score (mNSS)

At 24 hours after MCAO modelling, mNSS was applied for the evaluation of the neurological function of rats at different time‐points according to the previously established procedure.^27^ Tests included balance‐beam, walking, abnormal behaviour, sensory, tail suspension tests and loss of reflex, respectively. The evaluation criteria were light functional deficit (1‐6 points), moderate functional deficit (7‐12 points) and severe functional deficit (13‐18 points).

### 2.9 Cardiac perfusion and fixation of the brain samples

The state of consciousness, general behaviour and physical activity of the rats were observed before the experiment, after the anaesthesia, after the surgery, during and after the modelling, respectively. After 24 hours of mNSS, 12 rats in each group were randomly selected for anaesthesia followed by fixing on the surgical plate to open the thoracic cavity. Then, 20‐mL sterile syringe was carefully inserted into the left ventricle, while the right atrial appendage was cut and injected with sterile normal saline until the outflow liquid became clear. Thereafter, 4% paraformaldehyde (about 10‐20 mL) was added. When the liver and limbs of rats turned white and stiff, the brain tissues were extracted from rats, observed by naked eyes, fixed in paraformaldehyde, and slices into tissue sections.

### 2.10 Terminal deoxynucleotidyl transferase dUTP nick end labelling (TUNEL) staining

The brain tissues were dehydrated by gradient alcohol, cleared with xylene, embedded in paraffin and then followed by preparation of tissue sections. The tissue sections were then dewaxed by xylene for 10 minutes, soaked in 100%‐75% gradient alcohol, and distilled water, each for 1 minute. Tissue sections were then detached by Protease K at 37°C for 20 minutes and soaked in 0.3% H_2_O_2_ methanol solution for further 20 minutes at room temperature. After equilibrating the tissue sections, the terminal deoxyribonuclease reaction solution was prepared with 45 μL equilibration buffer, 5 μL nucleotide mixture, 1 μL terminal deoxyribonucleic acid transferase in each well. Thereafter, the slices were added with 50 μL terminal deoxyribonucleic reaction solution in each well for a 1‐hour reaction at 37°C. After incubation, the reaction was terminated at room temperature by adding 50 μL diluted 2 × saline sodium citrate (SSC) in dark. Subsequently, the tissue sections were stained with 4’6‐diamidino‐2‐phenylindole (1 μL/mL) at room temperature for 15 minutes followed by the application of an anti‐fade solution to the fluorescently labelled tissues sections (Cat#57461; Molecular Probe, Thermo Fisher Scientific). The brain tissues were photographed in different fields (six non‐overlapping, high‐resolution fields of view were randomly selected in each slice) with 488 nm and 405 nm under a fluorescence microscope, and the number of cells with apoptotic nuclei and total cells was counted. The apoptotic index (AI) indicated the number of apoptotic nuclei among 100 nuclei. AI = TUNEL‐positive cells/total cells × 100%.

### 2.11 BrdU staining

BrdU is commonly used to label newly synthesized DNA in living cells and can be selectively integrated into newly synthesized DNA in replicating cells instead of thymine. With DNA replication into daughter cells, BrdU‐specific antibody can detect the incorporation of BrdU, so as to judge the proliferation ability of cells. For this experiment, after BrdU solution was injected in vivo, the number of BrdU‐positive cells was detected by immunofluorescence to evaluate the proliferation ability of BrdU cells. BrdU (50 mg/kg; Sigma‐Aldrich) was administered on days 4, 5 and 6 after MCAO. The MCAO rats were perfused with 0.9% normal saline and 4% paraformaldehyde 2 weeks after the onset of MCAO. The brain was taken for immunohistochemistry. The sections were incubated in hydrochloric acid for 1 hour to denaturate the DNA and then neutralized with 0.1 M boric acid (pH 8.5) for 5 minutes twice. The number of BrdU‐labelled new cells was detected with anti‐BrdU antibody (anti‐BrdU antibody [BU1/75 (ICR1)] (Alexa Fluor^®^ 647) (ab220075)) according to the immunofluorescence procedure, and the number of positive cells was observed under fluorescence microscope (LSM510; Carl Zeiss MicroImaging).^28^

### 2.12 Immunohistochemistry

Immunohistochemistry staining was performed 2 or 4 weeks after MCAO. The slices were pretreated with citric acid buffer for 5 minutes, then incubated with 5% normal goat serum for 1 hour, incubated with primary antibody Ki‐67 (BD Pharmingen) as the marker of mitotic cells overnight at 4°C and then incubated with goat anti‐rabbit antibody (ab6721; Abcam Inc) for 30 minutes at 37°C. After PBS washing, the slices were incubated with horseradish‐labelled working solution, developed with DAB for 3‐10 minutes, restained with haematoxylin for 1 minute, sealed with neutral resin and photographed under the microscope. The staining time was adjusted under the microscope. ImageJ software was used to count and analyse the number of positive cells in tissue staining.^28^, ^29^

### 2.13 Enzyme‐linked immunosorbent assay (ELISA)

The IL‐9 content of the brain tissues in the experimental group and the control group was measured by an ELISA kit (R&D Systems). The 10% tissue homogenate (1000 μL) was centrifuged at 4000 rpm/min for 10 min at 4°C followed by the collection of the supernatant. Then, the standard samples were added with 2 mL of distilled water to prepare a 20 ng/mL standard sample solution. For this purpose, eight standard tubes were set, in which the first tube was added with a 900 μL diluted sample solution, while the rest of the tubes were added with a 500 μL sample solution. The content in each tube was repeatedly diluted with the eighth tube set as a blank control. Then, each well was added with 100 μL standard or test samples and placed on the reaction place at 37°C for 120 minutes. Each sample was detected following the instructions of the ELISA kit. The corresponding IL‐9 content was determined on the curve based on the sample optical density (OD) value.

### 2.14 Reverse transcription‐quantitative polymerase chain reaction (RT‐qPCR)

The total RNA was extracted by the TRIzol method, while the purity and concentration of the sample RNA were determined by a microplate reader (DNM‐9606; Beijing Pulang New Technology Co., Ltd). The total RNA was extracted using the RNeasy Mini Kit (Qiagen). The complementary DNA (cDNA) of miRNA was obtained by the reverse transcription of miRNA using the miRNA First Strand cDNA Synthesis (Tailing Reaction) kit (B532453‐0020; Sangon Biotech) and that of mRNA by the reverse transcription of mRNA using the reverse transcription kit (RR047A; Takara Bio), respectively.^9^ RT‐qPCR was performed on a Bio‐Rad instrument (10 021 337; Bio‐Rad). The relative mRNA expression was detected by real‐time fluorescent quantitative PCR using the SYBR Green (1 725 270; Bio‐Rad), while U6 was utilized for loading control of miR‐381, and glyceraldehyde‐3‐phosphate dehydrogenase (GAPDH) for other genes. The primer sequences are shown in Table 1. The expression levels of mRNA in brain tissues were calculated by the 2^‐ΔΔCt^ method.^30^

**Table 1:** Primer sequences for RT‐qPCR

	Primer sequences
miR‐381	F: 5'‐AGTCTATACAAGGGCAAGCTCTC‐3'
	R: 5'‐ATCCATGACAGATCCCTACCG‐3'
IRF4	F: 5'‐GCCCAACAAGCTAGAAAG‐3'
	R: 5'‐TCTCTGAGGGTCTGGAAACT‐3'
IL‐9	F: 5'‐TCTCTGATGCTGTTGCTGCT‐3′
	R: 5'‐CGTGGAACGGTTGAGGTAGT‐3′
U6	F: 5′‐GCATGACGTCTGCTTTGGA‐3'
	R: 5′‐CCACAATCATTCTGCCATCA‐3'
GAPDH	F: 5'‐ GACATGCCGCCTGGAGAAAC‐3'
	R: 5'‐AGCCCAGGATGCCCTTTAGT‐3'

Abbreviations: F, forward; GAPDH, glyceraldehyde‐3‐phosphate dehydrogenase; IL‐9, interleukin‐9; IRF4, interferon regulatory factor 4; miR‐381, microRNA‐381; R, reverse.

### 2.15 Dual‐luciferase reporter gene assay

The 3' untranslated region (UTR) dual‐luciferase reporter gene plasmids of IRF4 and the mutant plasmids of the miR‐381 binding site were constructed, namely pmirGLO‐IRF4‐wild‐type (WT) and PmirGLO‐IRF4‐mutant type (MUT), respectively. The reporter plasmids were separately cotransfected with miR‐381 mimic and mimic NC into the HEK293T cells. The cells were then lysed 24 hours after transfection and centrifuged at 12 000 rpm for one minute followed by the collection of the supernatant. Hence, the luciferase activity was detected using a Dual‐Luciferase Reporter Assay System (E1910; Promega).

### 2.16 Western blot analysis

Total protein was extracted from 10% brain tissues homogenate in the experimental group and the control group, respectively, and then centrifuged at 4°C and 500 rpm for 15 minutes, followed by the collection of the supernatant. The protein concentration was determined by the Brad‐ford method. Briefly, 30 μg sample protein was mixed well with immobilized pH gradient strip solution, while 10% sodium dodecyl sulphate‐polyacrylamide gel electrophoresis gel was introduced for 2‐hour electrophoresis. Then, the protein was transferred to the polyvinylidene fluoride membrane which was blocked by the skim milk powder at room temperature for 2 hours. The membrane was immersed in IRF4 polyclonal antibody (sc‐6059, 1:500; Santa Cruz Biotechnology), IL‐9 rabbit polyclonal antibody (ab205718, 1:500; Abcam Inc) and GAPDH monoclonal antibody (ab9485, 1:2000; Abcam Inc). Then, the membrane was rinsed with TBST for three times (10 min/each), incubated with horseradish peroxidase (HRP)–labelled goat anti‐rabbit immunoglobulin G (IgG) or goat antimouse secondary antibody (ab6721, 1:2000; Abcam Inc) for one hour, and developed using diaminobenzidine (DAB) liquid. Images were obtained using a gel imager (Gel Doc XR; Bio‐Rad). The grey value of the target band was determined using image analysis software ImageJ (National Institutes of Health), and the relative protein expression was described as the ratio of the grey value of the target protein to the internal reference protein band.

### 2.17 Flow cytometry

Flow cytometry was performed to detect the apoptotic rate of cells. Specifically, the brain tissues were cut, ground, trypsinized and rinsed with phosphate buffer saline. The 1 × 10^3^ cells/mL single cell suspension was prepared and stained with Annexin V‐propidium iodide (PI). After that, a flow cytometer (Gallios; Beckman Coulter Inc) was used to read the fluorescence at the excitation wavelength of 488 nm to detect cell cycle progression.

### 2.18 Statistical analysis

The SPSS 21.0 (IBM SPSS Statistics) was employed for statistical analysis. The measurement data were exhibited as the mean ± SD. Data comparisons among multiple groups were performed using one‐way analysis of variance (ANOVA), and the paired mean values among groups were compared with Tukey's post hoc test. Data among multiple groups at different time‐points were compared by repeated‐measures ANOVA followed by Bonferroni's post hoc test. Non‐parametric test (Mann‐Whitney U test or Kruskal‐Wallis test) was used to compare the neurological scores. The differences were statistically significant at *P* < .05.

## 3 RESULTS

### 3.1 Dex ameliorates cerebral ischaemic injury in rats by activating miR‐381

**Figure 1:**
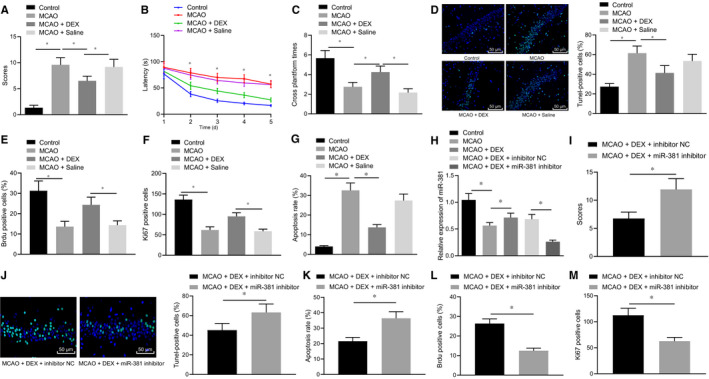
Dex enhances the miR‐381 expression to alleviate cerebral ischaemic injury in the MCAO rat. A, The mNSS score of rats after MCAO model establishment and Dex injection. B‐C, Latency for searching for the hidden platform and number of crossings the platform of rats after MCAO model establishment and Dex injection. D, TUNEL staining on brain tissues of rats after MCAO model establishment and Dex injection. E, BrdU‐positive cell rate was detected by BrdU. F, Ki‐67 staining to detect the number of Ki‐67‐positive expression. G, Flow cytometry analysis of cell apoptosis in brain tissues of rats after MCAO model establishment and Dex injection. H, miR‐381 expression of rats after MCAO model establishment, Dex injection and miR‐381 inhibitor treatment detected by RT‐qPCR (normalized to U6). I, The mNSS score of rats. J, TUNEL staining results of brain tissues of rats (scale bar = 100 μm). K, Apoptotic cells in brain tissues of rats measured by TUNEL staining. L, Cell apoptosis in brain tissues of rats determined by flow cytometry. N, BrdU‐positive cell rate was detected by BrdU. M, Ki‐67 staining to detect the number of Ki‐67‐positive expression. *P < .05. The above measurement data were expressed as the mean ± SD. The data of multiple groups were compared by one‐way ANOVA and Tukey's post hoc test. Data comparisons at different time‐points were performed by repeated‐measures ANOVA and Bonferroni's post hoc test. Non‐parametric test (Mann‐Whitney U test) was used to compare the neurological scores. n = 12

To investigate whether Dex could regulate cerebral ischaemic injury, the MCAO rat model was developed and injected with Dex followed by evaluation of the neurological functions by mNSS. We found that after induction of the MCAO model, the neurological function of rats was significantly impaired, indicating that the MCAO modelling was successful. On the other hand, Dex markedly improved the neurological injury caused by the MCAO (Figure 1A). While after Dex treatment, the neurological injury in the MCAO modelled rats was alleviated (Figure 1B). Thereafter, the learning and memory ability of rats was assessed by the water maze test and our results indicated that the MCAO rats spent increased time searching for the hidden platform, which was reversed by injection of Dex. The escape latency analysis of the hidden platform test revealed differences between the groups (Figure 1C). According to the TUNEL staining and flow cytometry results, Dex distinctly reduced the cell apoptosis caused by MCAO (Figure 1D, G). BrdU and Ki‐67 staining results showed that compared with the sham operated rats, the BrdU‐positive cell rate and Ki‐67‐positive expression number in the MCAO modelled rats were significantly reduced, and the BrdU‐positive cell rate and Ki‐67‐positive expression number were significantly increased after Dex treatment (Figure 1E, F).

To verify the protective effect of miR‐381 on the cerebral ischaemic injury, the miR‐381 inhibitor was introduced into MCAO rats injected with Dex. The results of RT‐qPCR demonstrated that the expression of miR‐381 was significantly reduced in MCAO rats, whereas injection of Dex elevated the miR‐381 expression in MCAO rats, which was reversed by additional treatment of miR‐381 inhibitor (Figure 1H). Moreover, the mNSS results exhibited that the neurological function deficit caused by the MCAO was improved by injection of Dex, which was abrogated by additional treatment of miR‐381 inhibitor (Figure 1I). While TUNEL staining and flow cytometry results further confirm that MCAO‐induced cell apoptosis was reduced by the injection of Dex, which was abolished by miR‐381 inhibitor (Figure 1J, K), BrdU and Ki‐67 staining results showed that compared with MCAO modelled rats treated with Dex and miR‐381 inhibitor NC, those treated with Dex and miR‐381 inhibitor had reduced BrdU‐positive cell rate and Ki‐67‐positive expression number (Figure 1L, M). Hence, the above‐mentioned results suggested that Dex was able to up‐regulate miR‐381 expression, thereby improving the cerebral ischaemic injury.

### 3.2 miR‐381 could bind to IRF4 to improve cerebral ischaemic injury in rats

To further investigate the downstream gene of miR‐381, the relevant bioinformatics analysis was conducted. A total of 4476, 3394, 2611 and 4150 downstream target genes of human miR‐381 were predicted using the StarBase, mirDIP, miRWalk and TargetScan, respectively. Further, 318 human transcription factors were obtained from the Cistrome database, which were intersected with the target genes of human miR‐381 predicted through bioinformatics analysis. And finally, 11 transcription factors (Figure S3A; Table S1) were obtained. The target genes of rat miR‐381 were predicted by the miRDB, miRWalk and TargetScan database, obtaining 403, 429 and 2225 downstream target genes, while fifteen target genes (Figure S3B; Table S2) were obtained by the intersection of the predicted results from the above three databases using the Venn map. Accordingly, our results indicated only the presence of transcription factor IRF4 in prediction results of both human and rat miR‐381. The binding sites between miR‐381 and IRF4 3'UTR are shown in Figure S3C.

**Figure 2:**
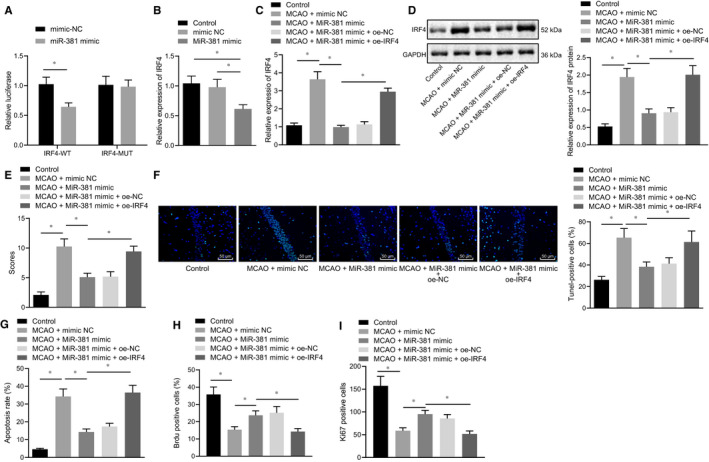
miR‐381 up‐regulation suppresses the cerebral ischaemic injury in MCAO rats by targeting the IRF4. A, Dual‐luciferase reporter gene assay on the binding relationship between miR‐381 and IRF4. B, IRF4 mRNA expression in primary neuron cells after miR‐381 overexpression detected by RT‐qPCR. C, IRF4 mRNA expression in MCAO rats after miR‐381 and IRF4 overexpression determined by RT‐qPCR. D, IRF4 protein levels in MCAO rats measured by Western blot analysis. E, Evaluation of neurological function on MCAO rats by mNSS. F, TUNEL analysis of apoptotic cells in brain tissues of MCAO rats (scale bar = 100 μm). G, Cell apoptosis in brain tissues of MCAO rats evaluated by flow cytometry. H, BrdU‐positive cell rate was detected by BrdU. I, Ki‐67 staining to detect the number of Ki‐67‐positive expression. *P < .05. The above‐reported measurement data were expressed as the mean ± SD. Data comparisons among multiple groups were performed using one‐way ANOVA and Tukey's post hoc test. Data comparison at different time‐points was performed by repeated‐measures ANOVA and Bonferroni post hoc test. Non‐parametric test (Kruskal‐Wallis test) was used to compare the neurological scores. n = 12. Each cellular experiment repeated three times

Subsequently, the targeting relationship between miR‐381 and IRF4 was verified by the dual‐luciferase reporter gene assay and the results indicated that the MUT‐IRF4 was the site of mutant S447 (S447A) and S448 (S448A), consistent with the previously reported work.^31^ Additionally, our results in (Figure 2A) showed that miR‐381 targeted and inhibited the IRF4 expression. Meantime, the cultured hippocampal primary neuron cells were treated with miR‐381 mimic. The results of RT‐qPCR showed the reduced expression of IRF4 in miR‐381 mimic‐treated primary neuron cells (Figure 2B). Similarly, the MCAO rats were treated with miR‐381 mimic and oe‐IRF4. Based on RT‐qPCR and Western blot analysis results, IRF4 expression was enhanced in MCAO rats. However, treatment with miR‐381 mimic declined IRF4 expression in MCAO rats, which was reversed by oe‐IRF4 treatment (Figure 2C, D).

Furthermore, the results of mNSS exhibited that miR‐381 mimic treatment decreased the neurological damage caused by MCAO, which was neutralized by the overexpression of IRF4 (Figure 2E). The results of TUNEL staining and flow cytometry demonstrated that miR‐381 mimic treatment significantly reduced the cell apoptosis induced by MCAO, which was reversed by the overexpressed IRF4 (Figure 2F, G). BrdU and Ki‐67 staining results showed that overexpression of miR‐381 significantly increased BrdU‐positive cell rate and Ki‐67‐positive expression number, while IRF4 overexpression restored the effect of miR‐381 (Figure 2H, I). Hence, the above‐mentioned results collectively demonstrated that miR‐381 inhibited the target gene IRF4 expression to ameliorate the cerebral ischaemic injury in MCAO rats.

### 3.3 Dex down‐regulates IRF4‐interleukin (IL)‐9 by activating miR‐381 in neuron cells

**Figure 3:**
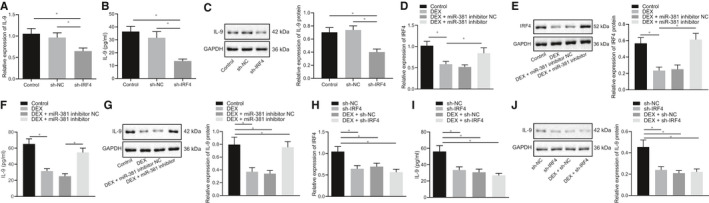
Dex up‐regulates the miR‐381 to repress the expression of IRF4‐IL‐9 in neuron cells. A‐C, The expression of IL‐9 in neuron cells after silencing IRF4 detected by RT‐qPCR, ELISA and Western blot analysis, respectively. D, E, The mRNA expression and protein level of IRF4 in neuron cells measured by RT‐qPCR and Western blot analysis, respectively. F, G, The expression of IL‐9 in cells treated with Dex and miR‐381 inhibitor studied using ELISA and Western blot analysis. H, The mRNA expression of IRF4 in neuron cells after treatment of si‐IRF4 and Dex measured by RT‐qPCR. I, J, The expression of IL‐9 in neuron cells after treatment of si‐IRF4 and Dex determined using ELISA and Western blot analysis. *P < .05. The above‐mentioned measurement data were expressed as the mean ± SD and analysed using one‐way ANOVA and Tukey's post hoc test. Each cellular experiment was repeated three times

Previous study has shown that the transcription factor IRF4 can promote the expression of IL‐9,^32^ so it is speculated that IRF4 may participate in the development of MCAO by inducing inflammatory reaction. To investigate the impact of IRF4 on IL‐9, IRF4 was silenced in primary neuron cells in vitro. Our data from RT‐qPCR, ELISA and Western blot analysis results indicated that IL‐9 expression was significantly decreased by silencing IRF4 in neuron cells (Figure 3A‐C), thus suggesting that IRF4 up‐regulated the IL‐9 expression.

To further verify whether Dex could directly or indirectly regulate the expression of IL‐9, primary neuron cells were cultured in vitro and treated with Dex and miR‐381 inhibitor. In the determination of RT‐qPCR and Western blot analysis, we found that IRF4 expression was reduced in neuron cells by the treatment of Dex, which was rescued by additional treatment of miR‐381 inhibitor (Figure 3D, E), indicating that the down‐regulation of IRF4 by Dex was dependent on the regulation of miR‐381. Then, IL‐9 expression was determined using ELISA and Western blot analysis, which showed that treatment of Dex resulted in a remarkable decline of IL‐9 expression in neuron cells, which was normalized by the additional miR‐381 inhibitor treatment. It suggested that the down‐regulation of IL‐9 by Dex can be achieved in a miR‐381‐dependent manner (Figure 3F, G). Moreover, primary neuron cells were then cultured and treated with sh‐IRF4 and Dex to confirm whether Dex regulated IL‐9 expression by relying on IRF4 or not. Accordingly, IRF4 expression was detected by RT‐qPCR and the results showed that treatment of si‐IRF4 or Dex significantly reduced IRF4 expression in neuron cells. However, in contrast to sh‐IRF4 neuron cells, IRF4 expression in cells treated with Dex + sh‐IRF4 was remarkably lowered (Figure 3H). Besides, our results from ELISA and Western blot analysis presented that IL‐9 expression was significantly decreased in cells after Dex or sh‐IRF4 treatment (Figure 3I, J), thus, suggesting that IL‐9 expression down‐regulated by Dex was dependent on IRF4. Collectively, these findings suggested that Dex could inhibit the IRF4‐IL‐9 by up‐regulating the miR‐381 in neuron cells.

### 3.4 Dex ameliorates cerebral ischaemic injury by reducing the inflammatory response induced by IRF4‐IL9

**Figure 4:**
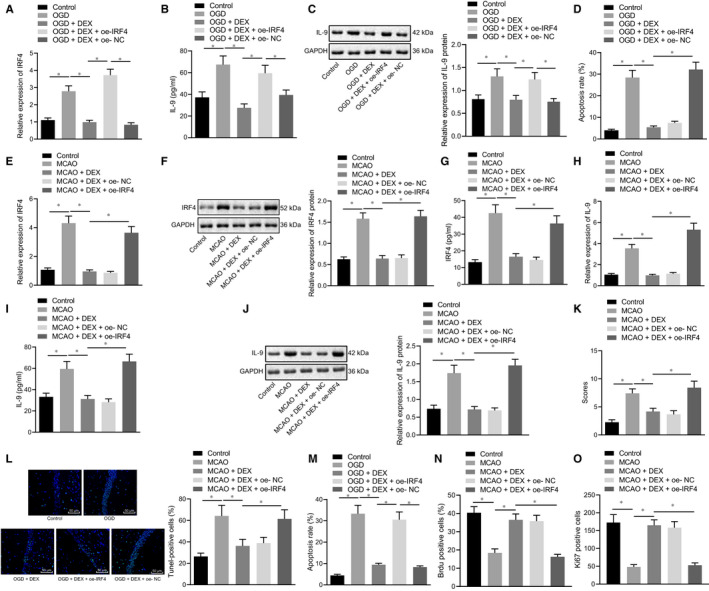
Dex reduces the inflammatory response by down‐regulating the IRF4‐IL‐9 to alleviate cerebral ischaemic injury. A‐C, The mRNA expression of IRF4 and IL‐9 in OGD‐treated neuron cells treated with Dex and oe‐IRF4 detected by RT‐qPCR, ELISA and Western blot analysis, respectively. D, Apoptosis of OGD‐treated neuron cells treated with Dex and oe‐IRF4 measured by flow cytometry. E‐G, The mRNA expression and protein expression of IRF4 in brain tissues of MCAO rats treated with Dex and oe‐IRF4 assessed by RT‐qPCR, ELISA and Western blot analysis, respectively. H‐J, The expression of IRF4 in brain tissues of MCAO rats observed by RT‐qPCR, ELISA and Western blot analysis. K, mNSS evaluation on the neurological function of MCAO rats. L, M, Cell apoptosis in brain tissues of MCAO rats detected by TUNEL staining (scale bar = 100 μm). N, Cell apoptosis in brain tissues of MCAO rats using flow cytometry. O, BrdU‐positive cell rate was detected by BrdU. P, Ki‐67 staining to detect the number of Ki‐67‐positive expression. *P < .05. The above‐mentioned measurement data were expressed as the mean ± SD, and compared using one‐way ANOVA and Tukey's post hoc test. Non‐parametric test (Kruskal‐Wallis test) was used to compare the neurological scores. n = 12. Each cellular experiment repeated three times

After validating the role of Dex in the regulation of IRF4‐IL‐9 expression *via* miR‐381, we further aimed to investigate its impact on cerebral ischaemic injury. For this purpose, the OGD experiment was performed on hippocampal primary neuron cells in vitro, in which OGD neuron cells were treated with Dex and oe‐IRF4. Our results from RT‐qPCR demonstrated that the highly expressed IRG4 was induced in cells by OGD treatment. However, Dex treatment reduced the IRF4 expression in OGD‐treated cells, which was rescued by the treatment of oe‐IRF4 (Figure 4A). Furthermore, our results from the ELISA and Western blot analysis illustrated that Dex reduced the highly expressed IL‐9 caused by OGD treatment in neuron cells, which was reversed by the overexpression of IRF4 (Figure 4B, C). According to flow cytometry, OGD‐treated neuron cells exhibited increased cell apoptosis, while Dex reduced apoptosis of OGD‐treated cells, which was reversed by the oe‐IRF4 treatment (Figure 4D).

Furthermore, the MCAO rats were treated with Dex and oe‐IRF4. The results of RT‐qPCR, Western blot analysis and ELISA assays showed that Dex reduced the IRF4 expression caused by MCAO, which was abrogated by the treatment of oe‐IRF4 (Figure 4E‐G). Meanwhile, the results of RT‐qPCR, ELISA and Western blot analysis detection on IL‐9 expression exhibited that Dex decreased the IL‐9 expression in MCAO rats, which was reversed by oe‐IRF4 treatment (Figure 4H‐J). Moreover, our results from the mNSS demonstrated that Dex improved the neurological impair caused by MCAO, while overexpression of IRF4 disabled this effect (Figure 4K). TUNEL and flow cytometry showed that Dex reduced cell apoptosis induced by MCAO, which was then neutralized by overexpressed IRF4 (Figure 4L‐N). BrdU and Ki‐67 staining results showed that after Dex treatment, BrdU‐positive cell rate and Ki‐67‐positive expression number were significantly increased compared with the MCAO group, while it was opposite after overexpression of IRF4 (Figure 4O, P). The aforementioned results cooperatively illustrated that Dex can improve the neurological impairment induced by MCAO, and overexpression of IRF4 may increase the neurological impairment induced by MCAO.

## 4 DISCUSSION

Insufficient flow of blood is considered as the major cause of cerebral ischaemic injury.^33^ While previously reported studies have revealed the multiple functions of Dex treatment, for instance, Dex could effectively improve the outcome of cardiac surgery.^34^ Intriguingly, Dex has been indicated to possess a neuroprotective role in rat cerebral ischaemic injury.^35^ The fact that accumulating studies have reported the beneficial effects of Dex on the cerebral ischaemic injury, yet to date, its underlying molecular mechanisms remained elusive. Thus, the present study unravelled that Dex could potentially induce highly expressed miR‐381, which further results in the inhibition of inflammation response in rats with cerebral ischaemic injury.

Initially, we found that Dex ameliorated the cerebral ischaemic injury in MCAO rats by up‐regulating miR‐381, accompanied by decreased cell apoptosis, improved learning and memorizing abilities, and neurological function. Accordingly, previous studies have verified the protective effect of Dex on neurological function.^36^ Moreover, Dex has also been indicated to improve the long‐term memory and learning ability in rats with the hypoxia/reoxygenation‐induced cerebral injury,^6^ while another study found that Dex could repair the brain ischaemic injury by promoting the extracellular signal‐regulated kinase‐related signalling pathway.^37^ Besides, it has been shown that the Dex administration elevated the expression of five miRNAs, including miR‐702‐3p, miR‐7a‐2‐3p, miR‐3596d, miR‐496‐5p and miR‐434‐3p in heart of rats, thus indicating the correlation between Dex and miRNAs.^38^ Additionally, in lung injury, Dex could elevate the expression of miR‐381 in modelled mice.^14^ While miR‐381 has reported to exhibiting protective effects on peripheral neuropathy.^39^ Thus, the repairing effect of miR‐381 in cerebral ischaemic injury has been validated before.^13^

Thereafter, we attempted to investigate, the downstream mechanism underlying Dex/miR‐38, and our data showed that overexpressed miR‐381 inhibited the transcriptional factor IRF4 expression to alleviate the cerebral ischaemic injury. Similarly, previous studies have shown the high expression of IRF4 in the mice with ischaemic brain injury,^40^ while another study demonstrated the significantly up‐regulated expression of IRF4 in rats with MCAO.^41^ Consistently, our results from Western blot analysis and RT‐qPCR determined the elevated expression of IRF4 in MCAO rats; however, it was declined after the addition of miR‐381 mimic, thus, reflecting that IRF4 was negatively correlated with miR‐381. Accordingly, a previous study has reported the overexpressed miRNA, namely, miR‐30b/d/e could notably inhibit the expression of IRF4 in plasma cell differentiation.^42^ Besides, miR‐125b has been reported to represses the IRF4 expression and results in the induction of B cell leukaemia and myeloid.^43^ Furthermore, our study revealed that IRF4 overexpression abolished the inhibitory effect of miR‐381 mimic on cell apoptosis and neurological function damage in MCAO rats. Additionally, it has been reported that IRF4‐positive is highly expressed in the ischaemic brain, which promoted the expression of inflammatory factor interleukin‐17.^44^

Finally, the most important finding of our study depicted that Dex reduced the inflammatory response by down‐regulating the IRF4‐IL‐9 via miR‐381 to alleviate cerebral ischaemic injury. Though previously reported study has shown the correlation between cerebral ischaemic injury, inflammation, cell apoptosis, and oxidation.^45^ Noticeably, accumulating studies have shown the role of IL‐9 in various types of inflammatory processes.^46^ Thus, IL‐9 has been verified as a potential pathogenic factor in ischaemic stroke.^47^ Besides, it has been also indicated that IRF4 promotes the expression of IL‐9 in a dose‐dependent way in human and mouse T helper nine cells.^32^ However, the participation of Dex could suppress the inflammatory response induced by lipopolysaccharide,^48^ while the anti‐inflammation effect of miR‐381 on macrophages has also been reported.^49^

## 5 CONCLUSION

**Figure 5:**
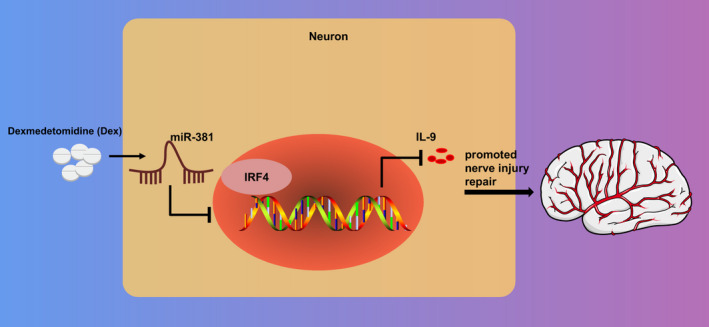
The mechanism of Dex in cerebral ischaemic injury with the involvement of miR‐381. The use of Dex induced highly expressed miR‐381, which led to an inhibition of inflammation response in rats with cerebral ischaemic injury by repressing IRF4‐IL‐9

In summary, our study suggested the protective effects of Dex exerts on cerebral ischaemic injury in vivo, which may account for its involvement in the regulation of inflammatory response by inhibiting IRF4‐IL‐9 via miR‐381 inhibitor (Figure 5). Intriguingly, the data presented in this study could be of clinical importance, thus, suggesting the therapeutic application of Dex as a neuroprotective agent. Nevertheless, the limitation remains. Additionally, the dosage of Dex used in the present study is applicable for rats only and cannot be used for the clinical practice. Therefore, further characterization of Dex‐mediated mechanisms is a prerequisite in future research.

## CONFLICT OF INTEREST

The authors declare no conflicts of interest.

## AUTHOR CONTRIBUTION

Hua Fang: Data curation (equal); Formal analysis (equal); Resources (equal). Hua‐Feng Li: Investigation (equal); Project administration (equal); Validation (equal). Jian‐Yong Yan: Supervision (equal); Writing‐original draft (equal). Miao Yang: Methodology (equal); Software (equal). Jian‐Ping Zhang: Conceptualization (equal); Funding acquisition (equal); Writing‐review & editing (equal).

## Supporting information

Fig S1

Fig S2

Fig S3

Table S1‐S2

## Data Availability

Research data are not shared.

## References

[1] **Li** S , **Jiang** D , **Rosenkrans** ZT , et al. Aptamer‐conjugated framework nucleic acids for the repair of cerebral ischemia‐reperfusion injury. Nano Lett. 2019;19:7334‐7341. 31518140 10.1021/acs.nanolett.9b02958 PMC6876547

[2] Singer BH . The vasculature in sepsis: delivering poison or remedy to the brain? J Clin Invest. 2019;130:1527‐1529. 10.1172/JCI127679 PMC6436847 30882365

[3] Cai W , Zhang K , Li P , et al. Dysfunction of the neurovascular unit in ischemic stroke and neurodegenerative diseases: an aging effect. Ageing Res Rev. 2017;34:77‐87. 27697546 10.1016/j.arr.2016.09.006 PMC5384332

[4] Salas‐Perdomo A , Miro‐Mur F , Urra X , et al. T cells prevent hemorrhagic transformation in ischemic stroke by P‐selectin Binding. Arterioscler Thromb Vasc Biol. 2018;38:1761‐1771. 29903733 10.1161/ATVBAHA.118.311284

[5] Giuliani D , Ottani A , Neri L , et al. Multiple beneficial effects of melanocortin MC4 receptor agonists in experimental neurodegenerative disorders: Therapeutic perspectives. Prog Neurogibol. 2017;148:40‐56. 10.1016/j.pneurobio.2016.11.004 27916623

[6] Gao Y , Yin H , Zhang Y , et al. Dexmedetomidine protects hippocampal neurons against hypoxia/reoxygenation‐induced apoptosis through activation HIF‐1alpha/p53 signaling. Life Sci. 2019;232:116611. 31260683 10.1016/j.lfs.2019.116611

[7] Abdel‐Ghaffar HS , Kamal SM , El Sherif FA , Mohamed SA . Comparison of nebulised dexmedetomidine, ketamine, or midazolam for premedication in preschool children undergoing bone marrow biopsy. Br J Anaesth. 2018;121:445‐452. 30032884 10.1016/j.bja.2018.03.039

[8] Wang L , Liu H , Zhang L , et al. Neuroprotection of Dexmedetomidine against Cerebral Ischemia‐Reperfusion Injury in Rats: Involved in Inhibition of NF‐kappaB and Inflammation Response. Biomol Ther (Seoul). 2017;25:383‐389. 27871154 10.4062/biomolther.2015.180 PMC5499616

[9] Mansouri F , Seyed Mohammadzad MH . Molecular miR‐19a in acute myocardial infarction: novel potential indicators of prognosis and early diagnosis. Asian Pac J Cancer Prev. 2020;21:975‐982. 32334458 10.31557/APJCP.2020.21.4.975 PMC7445987

[10] Zhou XM , Liu J , Wang Y , et al. microRNA‐129‐5p involved in the neuroprotective effect of dexmedetomidine on hypoxic‐ischemic brain injury by targeting COL3A1 through the Wnt/beta‐catenin signaling pathway in neonatal rats. J Cell Biochem. 2018;120(5):6908–6919. 10.1002/jcb.26704 29377229

[11] Huang Z , Liu G , Zeng Q , et al. MiR‐29b expression is associated with a dexmedetomidine‐mediated protective effect against oxygen‐glucose deprivation‐induced injury to SK‐N‐SH cells in vitro. Cell Biol Int. 2018;42:344‐352. 29087603 10.1002/cbin.10906

[12] Shi X , Yan C , Liu B , et al. miR‐381 regulates neural stem cell proliferation and differentiation via regulating Hes1 expression. PLoS One. 2015;10:e0138973. 26431046 10.1371/journal.pone.0138973 PMC4591969

[13] Piao JM , Wu W , Yang ZX , et al. MicroRNA‐381 favors repair of nerve injury through regulation of the SDF‐1/CXCR4 signaling pathway via LRRC4 in acute cerebral ischemia after cerebral lymphatic blockage. Cell Physiol Biochem. 2018;46:890‐906. 29669322 10.1159/000488821

[14] Zhang Y , Wang X , Liu Z , Yu L . Dexmedetomidine attenuates lipopolysaccharide induced acute lung injury by targeting NLRP3 via miR‐381. J Biochem Mol Toxicol. 2018;32:e22211. 30102002 10.1002/jbt.22211

[15] Hazra B , Kumawat KL , Basu A . The host microRNA miR‐301a blocks the IRF1‐mediated neuronal innate immune response to Japanese encephalitis virus infection. Sci Signal. 2017;10:eaaf5185. 28196914 10.1126/scisignal.aaf5185

[16] Al Mamun A , Yu H , Mirza MA , et al. Myeloid cell IRF4 signaling protects neonatal brains from hypoxic ischemic encephalopathy. Neurochem Int. 2019;127:148‐157. 30586599 10.1016/j.neuint.2018.12.014 PMC6579623

[17] Li JH , Liu S , Zhou H , Qu LH , Yang JH . starBase v2.0: decoding miRNA‐ceRNA, miRNA‐ncRNA and protein‐RNA interaction networks from large‐scale CLIP‐Seq data. Nucleic Acids Res. 2014;42:D92‐D97. 24297251 10.1093/nar/gkt1248 PMC3964941

[18] Tokar T , Pastrello C , Rossos AEM , et al. mirDIP 4.1‐integrative database of human microRNA target predictions. Nucleic Acids Res. 2018;46:D360‐D370. 29194489 10.1093/nar/gkx1144 PMC5753284

[19] Sticht C , De La Torre C , Parveen A , Gretz N . miRWalk: an online resource for prediction of microRNA binding sites. PLoS One. 2018;13:e0206239. 30335862 10.1371/journal.pone.0206239 PMC6193719

[20] Agarwal V , Bell GW , Nam JW , Bartel DP . Predicting effective microRNA target sites in mammalian mRNAs. Elife. 2015;4. 10.7554/eLife.05005 PMC4532895 26267216

[21] Mei S , Meyer CA , Zheng R , et al. Cistrome cancer: a web resource for integrative gene regulation modeling in cancer. Cancer Res. 2017;77:e19‐e22. 29092931 10.1158/0008-5472.CAN-17-0327 PMC5826647

[22] Chen Y , Wang X . miRDB: an online database for prediction of functional microRNA targets. Nucleic Acids Res. 2020;48:D127‐D131. 31504780 10.1093/nar/gkz757 PMC6943051

[23] Liu X , Wang Z , Wang P , et al. Green tea polyphenols alleviate early BBB damage during experimental focal cerebral ischemia through regulating tight junctions and PKCalpha signaling. BMC Complement Altern Med. 2013;13:187. 23870286 10.1186/1472-6882-13-187 PMC3723424

[24] Longa EZ , Weinstein PR , Carlson S , Cummins R . Reversible middle cerebral artery occlusion without craniectomy in rats. Stroke. 1989;20:84‐91. 2643202 10.1161/01.str.20.1.84

[25] Wang BX , Xu JJ , Hu J , et al. Effects of miR‐153 on angiogenesis in MCAO rats through Shh signaling pathway. Eur Rev Med Pharmacol Sci. 2019;23:732‐739. 30720181 10.26355/eurrev_201901_16887

[26] Niesman IR , Schilling JM , Shapiro LA , et al. Traumatic brain injury enhances neuroinflammation and lesion volume in caveolin deficient mice. J Neuroinflammation. 2014;11:39. 24593993 10.1186/1742-2094-11-39 PMC3975903

[27] Li Y , Chopp M , Chen J , et al. Intrastriatal transplantation of bone marrow nonhematopoietic cells improves functional recovery after stroke in adult mice. J Cereb Blood Flow Metab. 2000;20:1311‐1319. 10994853 10.1097/00004647-200009000-00006

[28] Belayev L , Hong SH , Menghani H , et al. Docosanoids promote neurogenesis and angiogenesis, blood‐brain barrier integrity, penumbra protection, and neurobehavioral recovery after experimental ischemic stroke. Mol Neurobiol. 2018;55:7090‐7106. 29858774 10.1007/s12035-018-1136-3 PMC6054805

[29] Liu Q , Jin Z , Xu Z , et al. Antioxidant effects of ginkgolides and bilobalide against cerebral ischemia injury by activating the Akt/Nrf2 pathway in vitro and in vivo. Cell Stress Chaperones. 2019;24:441‐452. 30815818 10.1007/s12192-019-00977-1 PMC6439064

[30] Li Y , Wang J , Zhou Y , Li D , Xiong ZQ . Rcan1 deficiency impairs neuronal migration and causes periventricular heterotopia. J Neurosci. 2015;35:610‐620. 25589755 10.1523/JNEUROSCI.1003-14.2015 PMC6605368

[31] Watanabe T , Asano N , Meng G , et al. NOD2 downregulates colonic inflammation by IRF4‐mediated inhibition of K63‐linked polyubiquitination of RICK and TRAF6. Mucosal Immunol. 2014;7:1312‐1325. 24670424 10.1038/mi.2014.19 PMC4177019

[32] Campos Carrascosa L , Klein M , Kitagawa Y , et al. Reciprocal regulation of the Il9 locus by counteracting activities of transcription factors IRF1 and IRF4. Nat Commun. 2017;8:15366. 28497800 10.1038/ncomms15366 PMC5437292

[33] Hou J , Yang X , Li S , et al. Accessing neuroinflammation sites: Monocyte/neutrophil‐mediated drug delivery for cerebral ischemia. Sci Adv. 2019;5:eaau8301. 31531392 10.1126/sciadv.aau8301 PMC6737273

[34] Ji F , Li Z , Nguyen H , et al. Perioperative dexmedetomidine improves outcomes of cardiac surgery. Circulation. 2013;127:1576‐1584. 23513068 10.1161/CIRCULATIONAHA.112.000936 PMC3979354

[35] Rodriguez‐Gonzalez R , Sobrino T , Veiga S , et al. Neuroprotective effects of dexmedetomidine conditioning strategies: evidences from an in vitro model of cerebral ischemia. Life Sci. 2016;144:162‐169. 26655164 10.1016/j.lfs.2015.12.007

[36] Zhao L , Zhai M , Yang X , et al. Dexmedetomidine attenuates neuronal injury after spinal cord ischaemia‐reperfusion injury by targeting the CNPY2‐endoplasmic reticulum stress signalling. J Cell Mol Med. 2019;23:8173‐8183. 31625681 10.1111/jcmm.14688 PMC6850922

[37] Teng L , Chen W , Yin C , Zhang H , Zhao Q . Dexmedetomidine improves cerebral ischemia‐reperfusion injury in rats via extracellular signal‐regulated kinase/cyclic adenosine monophosphate response element binding protein signaling pathway. World Neurosurg. 2019;127:e624‐e630. 30930327 10.1016/j.wneu.2019.03.232

[38] Wang L , Tang S , Wang Z , et al. The administration of dexmedetomidine changes microRNA expression profiling of rat hearts. Biomed Pharmacother. 2019;120:109463. 31541882 10.1016/j.biopha.2019.109463

[39] Lee JS , Kwak G , Kim HJ , et al. miR‐381 attenuates peripheral neuropathic phenotype caused by overexpression of PMP22. Exp Neurobiol. 2019;28:279‐288. 31138995 10.5607/en.2019.28.2.279 PMC6526106

[40] Zhao SC , Wang C , Xu H , et al. Age‐related differences in interferon regulatory factor‐4 and ‐5 signaling in ischemic brains of mice. Acta Pharmacol Sin. 2017;38:1425‐1434. 28905935 10.1038/aps.2017.122 PMC5672072

[41] Lin Y , Zhang L , Dai Y , et al. Expression of interleukin‐9 and its upstream stimulating factors in rats with ischemic stroke. Neurol Sci. 2015;36:913‐920. 25652434 10.1007/s10072-015-2096-2

[42] Kassambara A , Jourdan M , Bruyer A , et al. Global miRNA expression analysis identifies novel key regulators of plasma cell differentiation and malignant plasma cell. Nucleic Acids Res. 2017;45:5639‐5652. 28459970 10.1093/nar/gkx327 PMC5449613

[43] So AY , Sookram R , Chaudhuri AA , et al. Dual mechanisms by which miR‐125b represses IRF4 to induce myeloid and B‐cell leukemias. Blood. 2014;124:1502‐1512. 25006123 10.1182/blood-2014-02-553842 PMC4148772

[44] Gelderblom M , Gallizioli M , Ludewig P , et al. IL‐23 (Interleukin‐23)‐producing conventional dendritic cells control the detrimental IL‐17 (Interleukin‐17) response in stroke. Stroke. 2018;49:155‐164. 29212740 10.1161/STROKEAHA.117.019101

[45] Wang D , Wang Q , Chen R , et al. Exploring the effects of Gastrodia elata Blume on the treatment of cerebral ischemia‐reperfusion injury using UPLC‐Q/TOF‐MS‐based plasma metabolomics. Food Funct. 2019;10:7204‐7215. 31609374 10.1039/c9fo01729a

[46] Gerlach K , Weigmann B . The dichotomous function of interleukin‐9 in cancer diseases. J Mol Med (Berl). 2019;97:1377‐1383. 31396657 10.1007/s00109-019-01826-5

[47] Tan S , Shan Y , Wang Y , et al. Exacerbation of oxygen‐glucose deprivation‐induced blood‐brain barrier disruption: potential pathogenic role of interleukin‐9 in ischemic stroke. Clin Sci (Lond). 2017;131:1499‐1513. 28550144 10.1042/CS20170984

[48] Bao Y , Zhu Y , He G , et al. Dexmedetomidine attenuates neuroinflammation in LPS‐stimulated BV2 microglia cells through upregulation of miR‐340. Drug Des Devel Ther. 2019;13:3465‐3475. 10.2147/DDDT.S210511 PMC6781164 31631971

[49] Liu Y , Gao Y , Yang J , et al. MicroRNA‐381 reduces inflammation and infiltration of macrophages in polymyositis via downregulating HMGB1. Int J Oncol. 2018;53:1332‐1342. 29956737 10.3892/ijo.2018.4463

