# Supplementary material for: Dexmedetomidine‐up‐regulated microRNA‐381 exerts anti‐inflammatory effects in rats with cerebral ischaemic injury via the transcriptional factor IRF4
Source: J Cell Mol Med. 2020 Dec 13;25(4):2098–109. doi: 10.1111/jcmm.16153 (PMC7882963; doi:10.1111/jcmm.16153)
Supplement: Supplementary file 4 — Table S1‐S2 [file JCMM-25-2098-s004.docx]

**Supplementary Table 1** Summary of hsa-miR-381 target genes

| Target gene | Accession number | Full name | Alias | Function |
| --- | --- | --- | --- | --- |
| POU2F1 | NM_002697 | POU Class 2 Homeobox 1 | OCT1, NF-A1, OTF-1, Oct-1B | Transcription factor that activates the promoters of the genes for some snRNA and of genes such as those for histone H2B and immunoglobulins. |
| PAX6 | NM_000280 | Paired Box 6 | D11S812E, WAGR, AN2, ASGD5, FVH1, MGDA | Transcription factor with important functions in the development of the eye, nose, central nervous system and pancreas. |
| FOXO1 | NM_002015 | Forkhead Box O1 | FOXO1A, FKH1, FKHR | Transcription factor that is the main target of insulin signaling and regulates metabolic homeostasis in response to oxidative stress |
| VEZF1 | NM_007146 | Vascular Endothelial Zinc Finger 1 | DB1, ZNF161 | Possible transcription factor. Specifically binds to the CT/GC-rich region of the interleukin-3 promoter and mediates tax transactivation of IL-3. |
| SMARCB1 | NM_001007468 | SWI/SNF Related, Matrix Associated, Actin Dependent Regulator Of Chromatin, Subfamily B, Member 1 | BAF47, PPP1R144, SNF5L1, Sfh1p, HSNFS, HSNF5, INI1, SWNTS1, MRD15, RTPS1 | Core component of the BAF (hSWI/SNF) complex |
| RBPJ | NM_203283 | Recombination Signal Binding Protein For Immunoglobulin Kappa J Region | IGKJRB, RBPJK, IGKJRB1, CBF1, KBF2, AOS3 | Transcriptional regulator that plays a central role in Notch signaling |
| EBF1 | NM_024007 | EBF Transcription Factor 1 | COE1, OLF1, OE-1 | Transcriptional activator |
| **IRF4** | NM_002460 | Interferon Regulatory Factor 4 | LSIRF, NF-EM5, MUM1, SHEP8 | Transcriptional activator |
| FOXO3 | NM_201559 | Forkhead Box O3 | AF6q21, FKHRL1, FOXO3A, FOXO2, FKHRL1P2 | Transcriptional activator |
| ESR1 | NM_000125 | Estrogen Receptor 1 | NR3A1, ESR, Era, ER, ESTRR, ESRA | Nuclear hormone receptor |
| PRKDC | NM_001081640 | Protein Kinase, DNA-Activated, Catalytic Subunit | DNPK1, P460, HYRC1, XRCC7, IMD26 | Serine/threonine-protein kinase |

**Supplementary Table2** Summary of rno-miR-381 target genes

| Target gene | Accession number | Full name | Alias | Function |
| --- | --- | --- | --- | --- |
| Dcun1d4 | NM_001108359 | defective in cullin neddylation 1 domain containing 4 | RGD1310422 | - |
| Mitf | NM_001191089 | melanocyte inducing transcription factor | - | Basic helix-loop-helix-leucine zipper transcription factor |
| Fam107b | NM_001025034 | family with sequence similarity 107, member B | - | Tumor suppressor gene |
| Zfp266 | NM_001135018 | zinc finger protein 266 | Zfp426, Znf266, Zfp426l | May be involved in transcriptional regulation |
| Crebrf | NM_001277157 | CREB3 regulatory factor | RGD1310862 | Acts as a negative regulator of the endoplasmic reticulum stress response or unfolded protein response (UPR) |
| Pias1 | NM_001106829 | protein inhibitor of activated STAT, 1 | - | E3-type small ubiquitin-like modifier (SUMO) ligase |
| Elmod2 | NM_001109506 | ELMO domain containing 2 | - | Acts as a GTPase-activating protein (GAP) toward guanine nucleotide exchange factors |
| Lrrtm4 | NM_001134746 | leucine rich repeat transmembrane neuronal 4 | RGD1560707 | May play a role in the development and maintenance of the vertebrate nervous system |
| Atp6v1h | NM_001013929 | ATPase H+ transporting V1 subunit H | SFD, VMA13, CGI-11, SFDbeta, SFDalpha | Subunit of the peripheral V1 complex of vacuolar ATPase |
| Calhm2 | NM_001008306 | calcium homeostasis modulator family member 2 | Fam26b, RGD1308276 | Pore-forming subunit of a voltage-gated ion channel |
| Slc24a3 | NM_053505 | solute carrier family 24 member 3 | Nckx3 | Transmembrane ion transporter protein |
| Sipa1l2 | NM_001009704 | signal-induced proliferation-associated 1 like 2 | Spar2, Sersap2 | Rap GTPase-activating protein |
| **Irf4** | NM_001106108 | interferon regulatory factor 4 | - | Transcriptional activator |
| Ppm1l | NM_001107681 | protein phosphatase, Mg2+/Mn2+ dependent, 1L | - | Acts as a suppressor of the SAPK signaling pathways |
| Trak2 | NM_133560 | trafficking kinesin protein 2 | GRIF-1, Als2cr3 | May regulate endosome-to-lysosome trafficking of membrane cargo |
